# Learning from Excellence as the first step in Appreciative Inquiry: Experience from a tertiary level academic maternity unit

**DOI:** 10.15694/mep.2017.000005

**Published:** 2017-01-11

**Authors:** Mary Higgins

**Affiliations:** 1 UCD Perinatal Research Centre

**Keywords:** Learning Excellence Appreciative Inquiry

## Abstract

This article was migrated. The article was marked as recommended.

Too often in Medicine, and Medical Education, we focus on what has gone wrong - the complication in a patient, the student in difficulty. This is a reactive approach that focuses on weaknesses and negativity. In contrast, a new approach has been proposed focusing on strengths, excellence and prospective planning. Whether called "Learning from Excellence" (in the clinical sphere), "Safety II" (in risk management) or Appreciative Inquiry in Medical Education, the common approach is to start by identifying and celebrating the processes that work well. This commentary reviews our experience in a Learning from Excellence (LfE) project in a large tertiary level academic unit in Dublin. As well as identifying areas of clinical excellence, it was gratifying that areas of educational excellence were identified. These areas covered the spectrum of multidisciplinary care within the hospital from undergraduate to multidisciplinary advanced care. Presentation of these findings may inspire others to start the first steps of Appreciative Inquiry in Medical Education.

## Introduction

Having trained and worked hard for many, many years I finally was appointed to a permanent academic/clinical job a couple of years ago. The training system in Ireland is particularly long (eleven years from graduation to completion of specialist training) so the overwhelming feeling was actually of relief, with an undercurrent of excitement that finally I could set down roots, get grants, set up projects and develop educational initiatives. Like most things in life, it wasn't quite that easy. It turns out that grants for medical education projects are small, that student expectations are changing and that not everyone is grateful for on the job teaching. Working in the clinical job, once people knew I had a genuine interest in postgraduate education then that suddenly meant that I was responsible for roll out of new guidelines and protocols. The constant pull of the trifeca of clinical work, education and research was a definite challenge, but something happened that pulled all three areas together and made a difference. I read about a project called "Learning from Excellence" (Kelly, 2016), started the project, reviewed it and then read the new AMEE guideline on "Appreciative Inquiry" (Sandars, 2016). Sometimes when numerous signs point you in a direction, you have to follow.

I work in Obstetrics and Gynaecology. The last few years have not been easy for those working in maternity care. The high profile media coverage of adverse events, reduction in public confidence of the service and increasing complexity has provided many, daily, ongoing challenges for staff. On the ground it has resulted in increased frustration and burnout amongst both clinical and non-clinical staff. There is a real and heartfelt wish to continue to provide high quality evidence based care to women and their children, but the demands on the service and the expectation of perfection makes this a real challenge. As well as the clinical challenges, it affects the other sides of the triangle: in education we can be blamed for not teaching appropriate skills, and research is seen as a "non urgent, not important" aspect no matter how much we argue the opposite.

Similar to most areas of medical care, maternity has largely focused on a reactive approach to safety, now commonly known as a "Safety I" approach (Hollnagel 2014). It's crucial to identify adverse incidents and errors and to then implement adaptations to avoid their recurrence. This approach has achieved good results, but has limitations - staff may not receive feedback from adverse events, changes may not be effective and it may be difficult to provide evidence of lessons being learnt. In addition, there is increasing evidence of the negative effect on staff and organizations. While the patient is, and always be, the primary focus of concern after adverse outcomes, there is evidence that staff involved may also be affected (the "second victim") as well as the healthcare organization (the "third victim"). Second victims have been shown to have an increased rate of anxiety, depression, post-traumatic stress, clinical confidence and suicidal ideation (Sirriyeh 2010).

Preparing a grant application on the second victim, I came across an emerging approach to safety, "Safety II" (Hollnagel 2014), which is based on the concept of resilience - the ability of an organisation to adapt to changes in conditions- the "work-arounds" that we do every day in clinical care, education and research. Safety II is also based on the concept of learning from excellence- notice "everyday examples of good practice", where by seeking and studying groups or individuals that perform exceptionally well that best practice can be identified. Appreciative Inquiry is a fundamental part of this concept (Cooperrider 2001, AMEE). 

## Action

Inspired by a project within a paediatric intensive care unit in the United Kingdom (Kelly, 2016), we decided to run a whole hospital "Learning from Excellence" (LfE) pilot within one calendar month. The project began with a hospital wide information campaign and included both clinical and non-clinical members of staff. Email and face-to-face contact was made with all departments in order to inform them of the pilot, the evidence base supporting it and how to report examples of excellence. Forms were circulated requesting examples of both individuals and team excellence and there were weekly reminders during the month encouraging reporting.

As individuals and groups were identified, a "Gold Star" award was presented to the nominee(s) - these were both given directly to the nominee(s) and publically published on a notice board on the hospital canteen. If appropriate each nominated team/individual received a copy of the nomination form so that they could identify what was the circumstances of the specific area of excellence. Themes were identified by content analysis of forms. A hospital wide presentation was made at Grand Rounds in November, when all nominated teams and individuals were identified, and a short video on this presentation was made and uploaded to the hospital intranet for those who could not attend. Possible patient identifiers were removed in order to protect confidentiality. 

## Discovery

Eighteen teams and 27 individuals received nominations for LfE; four LfE themes were identified. The first three, as expected, related to clinical work. "Excellence in Daily work", was illustrated by examples from clinical and administrative teams. "Identifying problems, proactively solving them" illustrated multidisciplinary staff-led initiatives to combat issues of importance to patients and staff. The third theme "Emergency Care" used real life clinical scenarios where good communication and teamwork made a difference - staff were described as "excellent", "calm" and "unflappable", handover as "seamless", care was "compassionate" and "supportive". These three themes are important, and underline high quality clinical care. What really surprised me was the fourth theme, "Educational Initiatives", as for me this reflects the foundations of good clinical care - without education we cannot provide excellence in daily work, cannot identify problems and know how to solve them, cannot, in fact, even provide emergency care.

As a proto cynic it was heartening to read examples where opportunities were taken to teach on the ground. Examples were given from the postnatal ward, where two women were admitted with a history of epilepsy, and the midwives reviewed when and how to give emergency antiepileptic medications using a "one minute tutorial" formula, that we had introduced to the hospital a couple of years previously (so two joys there - the teaching and the use of the teaching tool). Again from the postnatal ward, a woman developed urinary retention, so the urogynaecology nurse specialist up skilled new staff on management of retention and intermittent self-catheterization. These real life educational initiatives will be more valuable to the staff when they have a woman in front of them that benefits directly from their increased knowledge.

There were other examples of excellence in Medical Education. The Medical Social Work department ran a national conference on Domestic Violence - turns out they had done this on an annual basis and no one else in the hospital knew. Cue multiple compliments and expressions of interest for future meetings from other members of the multidisciplinary team. The hospital management had introduced annual mandatory "Skills and Drills" (S&D) training three years previously, and it was getting harder to carve out time to organize everyone to attend. Following the LfE pilot, our enthusiasm has reawakened, as several staff members commented how much emergency care had improved following S&D. Many doctors saying that they would run to an emergency and the midwives had already started the essential and then advanced care of the woman or infant; the midwives commented how satisfying it was to be able to start and continue care until the rest of the team arrived.

The undergraduate students complimented the teaching programme for excellence, and in particular the labour ward staff (a common comment was "best week in medical school ever!"). Four years after starting, we finally received our first complements on the training programmes for new doctors on labour ward management and early pregnancy ultrasound. It reminded me of the scene in "School of Rock" where one of the students come to Jack Blacks character and compliments him on a "fantastic lesson" to the amazement and delight of the other school teachers (lets leave aside what he was actually teaching..). Appreciation is so important. 

## Final thoughts

The effect of the LfE programme on staff has been incredibly valuable, resulting in increased discussion of positive outcomes, and a commitment to recognize what has been done well and to continue to do so in order to constantly improve our high quality care. As an educator it was particularly heartening to see our work is being appreciated, and truly making a difference to clinical care. Sometimes in education you don't get that feedback, but this month we did. This project is the first step in the AI cycle - "discovery" - the identification of processes that work well. We have three other phases to complete - dream, design and deliver - but the start was wonderful. It is my sincere and heartfelt opinion that others should try this as well, and not just teach it, but do it also. 

## Take Home Messages

Appreciative Inquiry (AI) starts by discovering areas of excellence within Medical Education.

By its very nature this discovery may unearth projects that have previously been unrecognised in the general arena.

Clinicians and non-clinicians alike deserve appreciation for innovations that result in excellence.

Discovery allows appreciation and recognition as well as opening the gates for the next steps in AI. 

Learning from Excellence and AI provides a balance to the possible negative issues of work, and can re-inspire others to greatness. 

## Notes On Contributors

Mary Higgins works as an Obstetrician, Maternal Medicine subspecialist, Academic and Researcher in the National Maternity Hospital and University College Dublin. As a result of the LfE project her proto-cynicism has waned.

## Bibliography/References

Cooperrider, D.L. & Whitney, D 2001. "A positive revolution in change". In Cooperrider, D. L.; Sorenson, P.; Whitney, D. & Yeager, T. Appreciative Inquiry: An Emerging Direction for Organization Development. Champaign, IL: Stipes. pp. 9-29. 

Hollnagel E., Wears R.L. and Braithwaite J. From Safety-I to Safety-II: A White Paper. 2014 The Resilient Health Care Net: Published simultaneously by the University of Southern Denmark, University of Florida, USA, and Macquarie University, Australia. Retrieved from http://resilienthealthcare.net/onewebmedia/WhitePaperFinal.pdf on December 6th 2016 

Kelly N, Blake S, Plunkett A. 2016. Learning from excellence in healthcare: a new approach to incident reporting. Arch Dis Child 101:788-9

https://doi.org/10.1136/archdischild-2015-310021

Losada, M. 1999. The complex dynamics of high performance teams. Math and Comp Model 30(9-10), 179-192

https://doi.org/10.1016/S0895-7177(99)00189-2

Sandars J, Murdoch-Eaton D. Appreciative inquiry in medical education. Med Teach 2016 Nov 17:1-5

https://doi.org/10.1080/0142159X.2017.1245852

Sirriyeh R, Lawton R, Gardner P, Armitage G. 2010. Coping with medical error: a systematic review of papers to assess the effects of involvement in medical errors on healthcare professionals' psychological well-being. Qual Saf Health Care 19:e43

https://doi.org/10.1136/qshc.2009.035253

## Appendices

## Declarations

The author has declared that there are no conflicts of interest.

