# Supplementary material for: Learning from Excellence as the first step in Appreciative Inquiry: Experience from a tertiary level academic maternity unit
Source: MedEdPublish (2016). 2017 Jan 11;6:5. [Version 1] doi: 10.15694/mep.2017.000005 (PMC10885215; doi:10.15694/mep.2017.000005)
Supplement: Peer review history

# Reviewer report: Reviewer response for version 1

**DOI:** 10.21956/mep.19419.r28524

**Published:** 2019-03-27

**Authors:** Ken Masters

**Affiliations:** 1 Sultan Qaboos University

This review has been migrated. The reviewer awarded 3 stars out of 5 An enjoyable and interesting paper. The author is correct in pointing out that we tend to focus on things that have gone wrong. This is for good reason, as the aim is to identify the errors, and then to create a situation in which these errors do not re-occur. It is common practice in all professions (it is how NASA moved from the disastrous Apollo 1 to the triumphant Apollo 11, and it is the feedback that our students want when they come to us after exams).As the author notes, however, if this is the only feedback that happens, then the long-term impact can be extremely harmful, and so it is necessary to look also at the successes, not only to celebrate them, but also to analyse why and how the success occurred.I think the author should, however, make the point more strongly that the idea is to find a balance, rather than have the pendulum swing the other way, otherwise the result will be ignoring of problems. Also, when analysing successes only, one runs the risk that “survivor bias” will lead to unreasonable expectations of success in the future, so safeguards against this need to be put into place.I would like to see elements of this approach formally built into teaching modules in the future. Perhaps the author would consider a follow-up paper on the topic.

Reviewer Expertise:

NA

No decision status is available

# Reviewer report: Reviewer response for version 1

**DOI:** 10.21956/mep.19419.r28526

**Published:** 2017-01-19

**Authors:** Mohammed Elhassan

**Affiliations:** 1 SAMP

This review has been migrated. The reviewer awarded 4 stars out of 5 Interesting experience and congratulations on the encouraging results that your facility noticed by implementing this project. As an academic hospitalist involved in teaching residents and medical students in internal medicine, I think this principle of “learning from excellence” is probably underutilized and we need to apply it more in our daily practice. To maintained sustainability of projects like this one is always challenging and needs continuous efforts to evaluate and re-evaluate such interventions on regular intervals and to look, not only into the positive results, but also to for any unexpected drawbacks that might be revealed in the future.

Reviewer Expertise:

NA

No decision status is available

# Reviewer report: Reviewer response for version 1

**DOI:** 10.21956/mep.19419.r28525

**Published:** 2017-01-11

**Authors:** Trevor Gibbs

**Affiliations:** 1 AMEE

This review has been migrated. The reviewer awarded 4 stars out of 5 Although I probably don't agree with the author that we concentrate too much on "what has gone wrong" - indeed we probably don't do that enough- I do agree that we need to strongly advertise the fantastic "little but important" things that happen everyday in the chaotic life of healthcare. This paper is a string advocate for seeking out and shouting about the good things that happen in healthcare education, in this case in a hospital setting, but equally could apply to other settings. I read this opinion / research paper with ease and fully understood the message, obviously relayed with passion for the subject- whatever you wish to call this activity, I feel that it represents an important step forward in motivating the positivity of medical education.

Reviewer Expertise:

NA

No decision status is available
